# Stated patient preferences for overnight at-home diagnostic assessment of sleep disorders

**DOI:** 10.1007/s11325-024-03080-7

**Published:** 2024-06-15

**Authors:** Marcel Braun, S Dietz-Terjung, U Sommer, C Schoebel, C Heiser

**Affiliations:** 1 West German Lung Center Essen, Center of Sleep and Telemedicine, University Hospital Essen, Tueschener Weg 40, 45239 Essen, Germany; 2 Faculty for Sleep and Telemedicine, University Duisburg-Essen, Essen, Germany; 3 Department for Otorhinolaryngology, Technical University Munich, Munich, Germany; 4 ENT Center Mangfall / Inn, Mangfall / Inn, Germany

**Keywords:** Sleep disorders, Diagnostic assessment, Overnight sleep testing, Patient preferences, Choice analysis, Discrete choice experiment

## Abstract

**Purpose:**

The diagnostic workup for assessment of sleep disorders commonly involves overnight testing to assess sleep patterns and pathological events. So far, little is known about preferences for provision of home sleep tests to patients with sleep disorders. This study aims to close this gap by eliciting preferences for home sleep testing using a discrete choice experiment (DCE).

**Methods:**

A DCE with seven attributes of at-home sleep testing and three levels per attribute was developed using a fractional factorial design. Patients with and without previous sleep testing experience were recruited from two large sleep centers in Germany. Coefficients for attribute levels were calculated using a conditional logit model to estimate their influence on choice decisions and calculate the relative importance of each attribute.

**Results:**

305 patients (54.5 ± 13,1 years, 65.3% male) were enrolled, and 288 surveys with complete data included for analysis. Attributes with greatest relevance were *Waiting time to discuss sleep study results*; *Waiting time to conduct sleep study*, and *Sleep quality during measurement*. Of lowest importance was *Diagnostic accuracy of sleep study*, followed by *Effort to apply sleep study device*. Significant heterogeneity in choice behavior was found, including differences by gender, willingness-to-pay for sleep studies, and previous experience with sleep studies. Preferred location for conducting sleep testing was at-home in 50.7% and in-lab in 46.9%.

**Conclusions:**

Preferences and relative importance of home sleep test attributes vary among different subgroups. Considering those preferences can be important for clinicians and policymakers when designing care pathways and planning of testing policies for sleep disorders.

**Supplementary Information:**

The online version contains supplementary material available at 10.1007/s11325-024-03080-7.

## Introduction

Sleep disorders leading to a disruption of sleep are increasingly recognized as significant contributors to a myriad of health issues, impacting the quality of life, overall well-being and long-term health of individuals [1]. Based on current research it is estimated that up to 40% of adults suffer from poor sleep or a sleep disorders, with snoring, obstructive sleep apnea, insomnia and restless-legs syndrome being the most common diseases [2–4]. Diagnosing these disorders commonly involves overnight sleep testing, which is crucial to understand sleep patterns and identify potential abnormalities and pathologic events. The current landscape of sleep testing encompasses a variety of methods, including in-laboratory polysomnography (PSG) and home sleep apnea testing (HSAT), which represent the most common methods of overnight sleep testing [5]. While in-laboratory PSG requires admission to a hospital or sleep center and is conducted with supervision of a trained PSG-technician, HSAT or polygraphy is commonly performed at the patients’ home without any surveillance. Though HSAT can be equivalent to an in-laboratory PSG in certain patient populations, this test does not provide direct measures of sleep and cannot be used for differential diagnosis of sleep disorders and hence, HSAT is limited to diagnosis of sleep apnea [6]. Accelerated by the SARS-CoV-2 pandemic, which limited access to in-laboratory PSG due to infection control measures, and enabled by technological developments, overnight sleep testing is increasingly shifting from the sleep laboratory to the patients’ home [5, 7]. With the introduction of modern sleep testing systems, comprehensive sleep test such as home-based PSG have the potential to extend the diagnostic portfolio of sleep medicine. This development is driven by constantly rising demand for sleep testing, as well as decreasing availability of skilled technical staff and rising hospital costs, which makes it increasingly difficult for patients to access overnight PSG testing.

As the field of sleep medicine continues to evolve, it becomes imperative to consider not only the efficacy of sleep diagnostic procedures but also the patient perspective and preferences associated with these tests. Current overnight sleep testing modalities differ not only in their diagnostic capabilities, but also in their convenience, cost, and overall experience they offer to patients. Understanding the factors that influence patient preferences in this context is paramount for tailoring diagnostic approaches that are not only clinically effective but also patient-centered and well accepted [8]. In absence of any evidence on this topic, this study aims to elicit the relevance of attributes of overnight sleep testing and preferences from the patients’ perspective.

## Methods

In general, elicitation of preferences, choice behavior and decision-making can be accomplished using two different approaches: revealed preferences, in which actual choices are observed and components of decision-making analyzed ex-post, after a good or service is obtained; and stated preferences, where decision-making is assessed ex-ante, before the consumption takes place, using theoretical choice tasks [9]. Since healthcare services, such as diagnostic tests or medical interventions, are commonly regulated by market authorizations, coverage policies and other insurance provisions, a revealed preference-approach can only be applied to assess choice behavior for technologies that are available and accessible by the respective patient population [10]. Alternatively, stated preference techniques can be used to elicit preferences for hypothetical technologies or healthcare scenarios, and furthermore, allow to identify attributes relevant to the choice decision before the actual decision is made. This enables decision-makers to design patient-centric healthcare services based on requirements and preferences before they are implemented. Various studies have shown, that advanced stated preferences techniques, such as discrete choice experiments (DCE), have a high external validity and can predict choice behavior well [11, 12]. As such, DCE are considered the standard methodology for elicitation of preferences and choice behavior in healthcare, and are thousands of studies have been conducted over the last decades [13].

### Experimental design & survey construction

To design a DCE, identification of relevant attributes of the technology or intervention subject to evaluation is required. In absence of any relevant literature on this topic, a set of attributes and levels that reflect the situation in the German sleep care environment was developed with input from three board certified sleep physicians. The attributes were then validated by a group of patients with various sleep disorders. The final set consisted of the attributes *Diagnostic accuracy* of sleep testing device, personal *Costs* per test in the form of a co-payment, *Effort to apply device* at home, *Logistics* of receiving and returning the diagnostic device, *Sleep quality during test, Waiting time to test*, and *Waiting time to discuss results* with the physician. All presented attributes and levels were considered relevant and appropriate by patients and hence the entire set was included in the experimental design (Table 1). To reduce the burden to participants of the study, a d-optimal fractional factorial design was applied, in line with the methodological recommendations for choice analysis using DCE, which decreases the number of choice tasks presented to each patient [14, 15]. Forced choice was applied in order to maximize information generation from the DCE.

**Table 1:** Attributes and respective attribute levels of overnight at-home sleep testing included in choice tasks

Attribute	Level 1	Level 2	Level 3
Diagnostic accuracy	Very good	Good	Acceptable
Costs	0€	100€	200€
Effort to apply device	Self-appliable, within 5 min.	Self-appliable, within 15 min.	With help, within 15 min.
Logistics	Receive via mail, return via mail	Receive from clinic, return via mail	Receive from clinic, return to clinic
Sleep quality during test	9 / 10 patients report good sleep quality	7 / 10 patients report good sleep quality	5 / 10 patients report good sleep quality
Waiting time to test	1 week	2 weeks	8 weeks
Waiting time to discuss results	Discussion with physician next morning after test	Discussion with physician within 48 h after test	Discussion with physician within 2 weeks after test

In addition to the DCE part, the survey included demographic information, self-reported symptoms of sleep disorders, prior experience, and items on Willingness-to-pay (WTP) and preferred sleep study location. The survey was administered in German language, and questions, choice tasks and explanations were optimized for patient understanding.

### Data collection

Participants were enrolled from two university hospitals in Germany that provide tertiary care to patients with sleep disorders and offer the entire range of sleep medical diagnostic and therapeutic services. Patients presenting to the outpatient department of the clinics with a suspected or confirmed diagnosis of a sleep disorder were invited to participate in the study. Most were referred to the centers by general practitioners or external specialists, which sent requests for consultations and patients commonly receive the first appointment within two to four weeks, in which the diagnostic pathway is defined. After providing written informed consent, participants received the survey in paper formed and provided their answers during their visit to the outpatient department, before they received any information about the planned diagnostic workup to avoid biasing their decision-making. The local ethics committees of both centers approved the study before patients were enrolled.

### Data management and analysis

Survey data was collected by the two centers and aggregated centrally for analysis. SPSS software (IBM, New York/USA) was used for data management as well as descriptive statistical analyses. R software with RStudio (r-project.org) was used for analysis of choice data from the DCE. Depending on research questions, hypotheses formulation and data distribution, a range of statistical tests was used, incl. Student’s t-test, Welch-test, Kruskal-Wallis test and Pearson Chi^2^ test. For management and analysis of DCE data, methodological recommendations from the International Society for Pharmacoeconomics and Outcome Research (ISPOR) were followed [14, 16]. The Wald test was conducted to evaluate the significance of attributes in the choice decisions made by participants [17]. Alpha levels of < 0.050 were considered statistically significant throughout all statistical analyses.

## Results

### Participants characteristics

From the two centers, a total of 305 consecutive patients were enrolled into the study. From those 305 participants, 288 questionnaires (94.4%) were completed sufficiently and could be included in the final analysis. The remaining 17 patients had to be excluded due to invalid DCE data. Reasons for invalid DCE data and thus exclusion were: DCE not completed; DCE only partially completed; and multiple choices where only single choices were allowed. No statistically significant differences were identified between the characteristics of the total population and patients with complete data sets (Table 2). Patients were predominantly male (65.3%) and on average 54.4 ± 13.1 years old (median = 55.0 years). Most patients reported symptoms of sleep-disordered breathing such as snoring / apneas (83.3%) or daytime sleepiness (23.6%). Other common symptoms mentioned were nightly awakening (54.2%), difficulties falling asleep (21.5%) or restless legs during sleep (19.4%). The majority of participants reported suffering from one (35.4%) or two symptoms (32.3%). The part of the sample which reported symptom snoring / apneas was quite heterogenous, with 51.4% reporting additional frequent nightly awakenings, 24.1% additional daytime sleepiness and 18.8% restless legs. Prior exposure to overnight sleep testing was common, with only 10.4% of patients not having received a sleep study before participating in the study. Of those with previous sleep study experience, HSAT was the most common overnight sleep test, (42.7%) followed by in-laboratory PSG (31.6%). Past experiences with both HSAT and in-laboratory PSG were reported by 15.3% of participants.

**Table 2:** Characteristics of total sample vs. included sample with complete data sets (SD = standard deviation, HST = Home Sleep Test, PSG = Polysomnography)

	Total sample (*n* = 305)			Included sample (*n* = 288)			
	%	Mean	± SD	%	Mean	± SD	*p*-value
Gender							0.452
Female	34.7			38.4			
Male	65.3			61.6			
Age (years)		54.4	13.1		54.4	13.1	0.392
Prior exposure to overnight sleep testing							
HST	41.6			42.7			0.396
In-lab PSG	32.5			31.6			0.411
HST and In-lab PSG	14.8			15.3			0.429
None	11.1			10.4			0.392
Self-reported symptoms							
Snoring or apneas	83			78.7			0.455
Nightly awakening	52.8			54.2			0.136
Daytime sleepiness	23.3			23.6			0.46
Difficulty falling asleep	21.6			21.5			0.488
Restless legs	20			19.4			0.433
Other	12.5			12.8			0.647
Number of symptoms reported							0.42
1 symptom	35.7			35.4			
2 symptoms	31.8			32.3			
3 symptoms	20.7			20.1			
˃3 symptoms	9.2			9.7			

**Table 3:** Coefficients for presented attributes of sleep testing, derived from conditional parameter logit model (log-likelihood = 182.8, AIC = 4216.39)

Attribute / level	Coeff.	SE	Z	*p*	Level diff.
*Diagnostic accuracy*					0.245
L1 - Very good	0.064	0.079	0.812	0.417	
L2 - Good	0.09	0.078	1.157	0.247	
L3 - Acceptable	-0.155	0.079	-1.95	0.051	
*Costs*					0.437
L1–0€	0.163	0.078	2.08	0.038	
L2–100€	0.112	0.078	1.434	0.151	
L3–200€	-0.274	0.078	-3.51	< .001	
*Effort to apply device*					0.183
L1 - Self-appliable, 5 min.	-0.183	0.08	-2.289	0.022	
L2 - Self-appliable, 15 min.	-0.062	0.083	-0.741	0.459	
L3 - With help, 15 min.	0.244	0.079	3.06	0.002	
*Logistics*					0.427
L1 - Receive via mail, return via mail	-0.124	0.079	-1.583	0.113	
L2 - Receive from clinic, return via mail	0.276	0.078	3.538	< .001	
L3 - Receive from clinic, return to clinic	-0.151	0.079	-1.92	0.054	
*Sleep quality during test*					0.549
L1–9 / 10 report good sleep quality	0.021	0.079	0.27	0.787	
L2–7 / 10 report good sleep quality	0.264	0.077	3.426	0.001	
L3–5 / 10 report good sleep quality	-0.285	0.079	-3.59	< .001	
*Waiting time to test*					0.519
L1 - Discussion with physician next morning	0.338	0.08	4.214	0.936	
L2 - Discussion with physician within 48 hours	-0.181	0.084	-2.156	< .001	
L3 - Discussion with physician within 2 weeks	-0.157	0.08	-5.48	< .001	
*Waiting time to discuss results*					0.893
L1–1 week	-0.007	0.081	-0.081	< .001	
L2–2 weeks	0.45	0.077	5.873	0.031	
L3–8 weeks	-0.443	0.801	-1.96	0.505	

### Discrete choice experiment

**Fig. 1:**
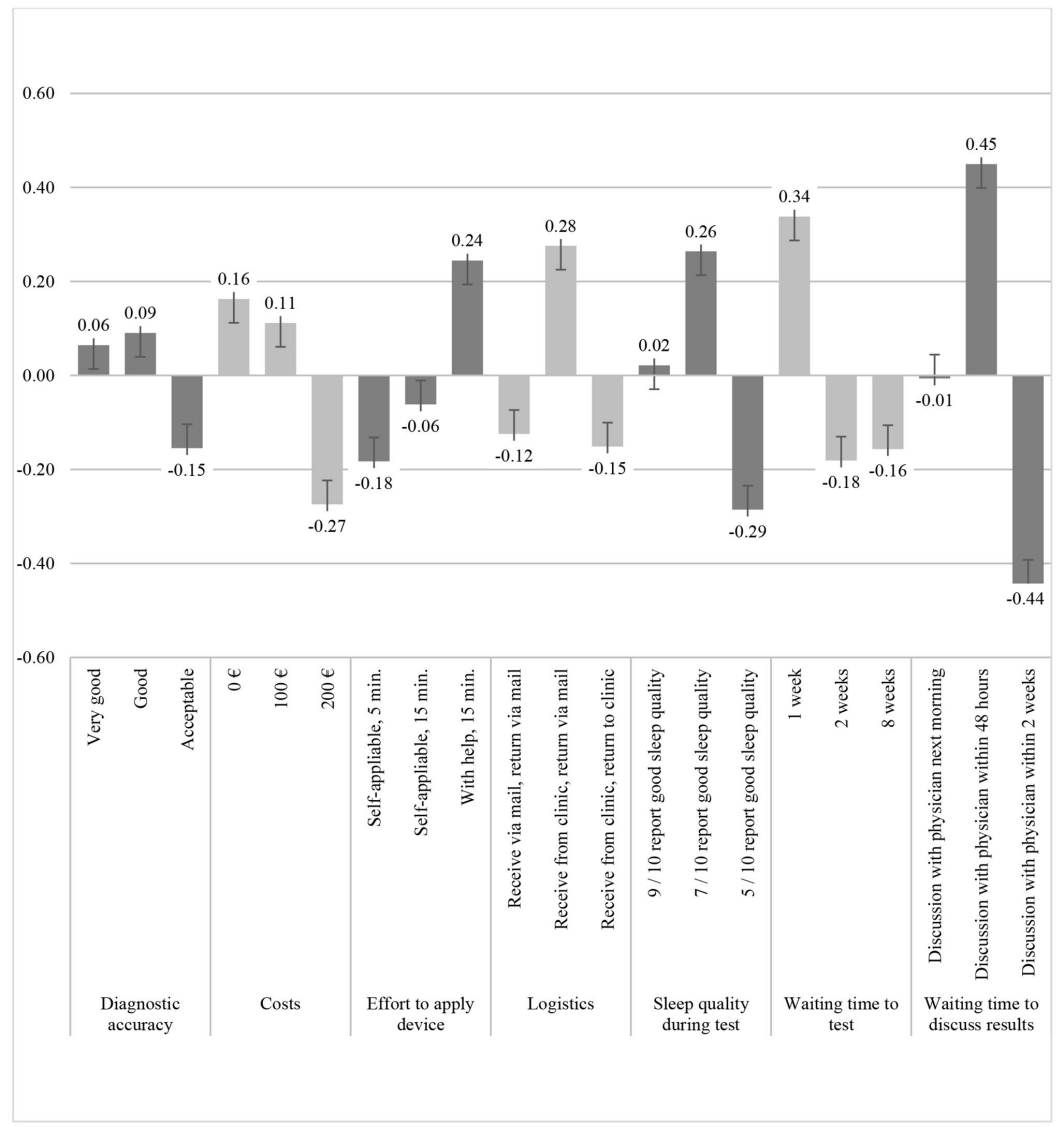
Coefficients of attribute levels (positive values indicate higher likelihood to choose alternative, negative values indicate lower likelihood to choose alternative)

DCE data was analyzed using a conditional logit model, from which coefficients for each attribute level were computed, which all showed the expected directions (Table 3) The Wald test was highly significant (97.92 (df = 14), *p = <* 0.001), suggesting the parameters used in the model provide significant prediction of the outcomes calculated. All alternatives presented in the choice experiment were statistically significant, except *Diagnostic accuracy*. This should be interpreted as this attribute not having a relevant effect on the choice decisions made by participants. Coefficients of attribute levels, shown in Table 3, represent the marginal utility of each alternative in the choice decision. Positive coefficients have a positive contribution to the choice decision and hence increase the probability that an alternative was chosen due the higher utility assigned to the attribute (Fig. 1). Contrary, negative coefficients have a negative influence on the decision and decrease the probability that an alternative was preferred by participants due to its lower utility.

**Fig. 2:**
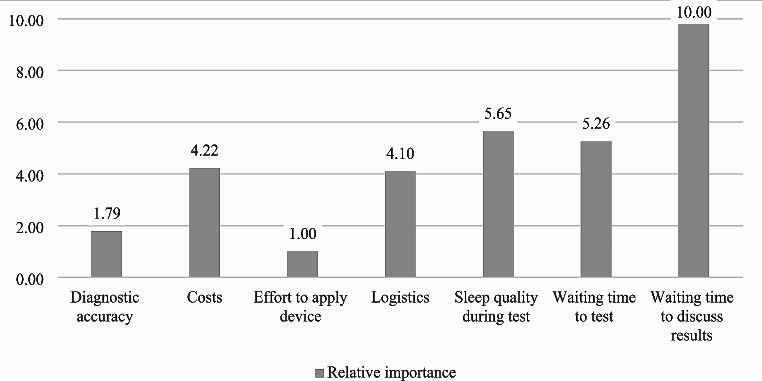
Relative importance of attributes of overnight at-home sleep testing, re-scaled from 0 (least important) to 10 (most important), all participants

The most relevant attributes for choice decisions were *Waiting time to discuss results*, with a coefficient of 0.893, followed by *Sleep quality during test* and *Waiting time to test* with coefficients of 0.549 and 0.519 respectively. *Diagnostic accuracy* and *Effort to apply diagnostic device* were of least importance (coefficients 0.245 and 0.183). Figure 2 presents the relative importance of each attribute in relation to the others, rescaled from 0 (least important) to 10 (most important).

In the DCE, the most preferred at-home overnight sleep test consisted of the following attributes: *Diagnostic accuracy* = good; Costs = 0€; *Effort to apply the device* = With help within 15 min; *Logistics* = received from clinic, return via mail; *Sleep quality* = 7/10 report good sleep quality; *Waiting time to test* = 2 weeks; *Waiting time to discuss results* = Discussion with physician next morning.

### Preferred sleep study location

**Fig. 3:**
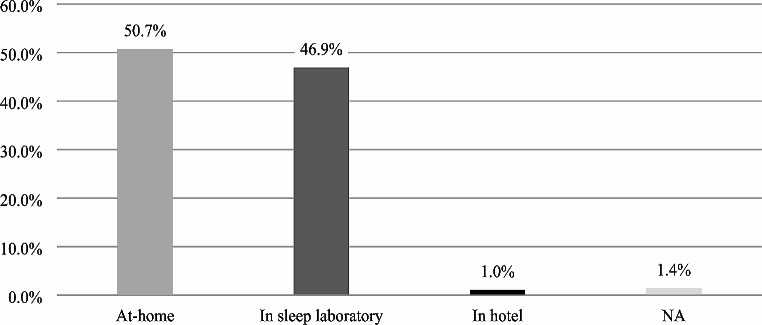
Preferred location for overnight sleep testing

Among patients enrolled in the study a preference of at-home sleep testing (50.7%) over testing in a sleep laboratory (46.9%) was found (Fig. 3). Preference of sleep testing in a hotel room was low with only 1.4% of participants choosing this option. Stratification by age, gender and prior sleep testing experience revealed significant differences in choices, and younger patients (≤ 55 years = 58.4% vs. ˃ 55 years = 43.1%), as well as male patients (male = 54.8% vs. female = 43.0%) and participants without prior experience (without experience = 71.0% vs. with experience = 48.2%) reported higher preferences for at-home testing. Further analysis revealed that patients with a preference for at-home overnight sleep testing were on average 5.6 years younger than those that preferred testing in a sleep laboratory (*p = <* 0.001).

### Willingness-to-pay

**Fig. 4:**
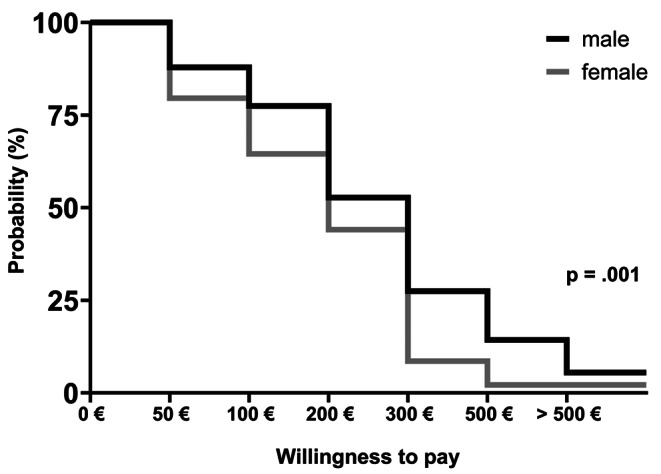
Willingness-to-pay out of pocket for overnight sleep testing, male vs. female participants

Overall WTP was high with 52.4% of the sample stating to spend 100–200€ out-of-pocket for overnight sleep testing, and 25.0% 200–300€ (Fig. 4). Male participants had a significantly higher WTP than female participants (*p = <* 0.001). The WTP of patients with a location preference of at-home testing was higher as well, compared to those with a preference for testing in a sleep laboratory (*p* = < 0.001). No differences were found between patients with and without prior sleep testing experience (*p =* .625). Additional analysis revealed a statistically significant correlation between WTP and symptoms reported, with lower number of symptoms correlating with lower WTP (*r* = -.132 (95% CI = -0.247 to -0.014); *p* = .028). No correlation was found between WTP and age (*r* = .006 (95% CI = -0.113 to 0.125); *p* = .921).

### Subgroup analyses

**Fig. 5:**
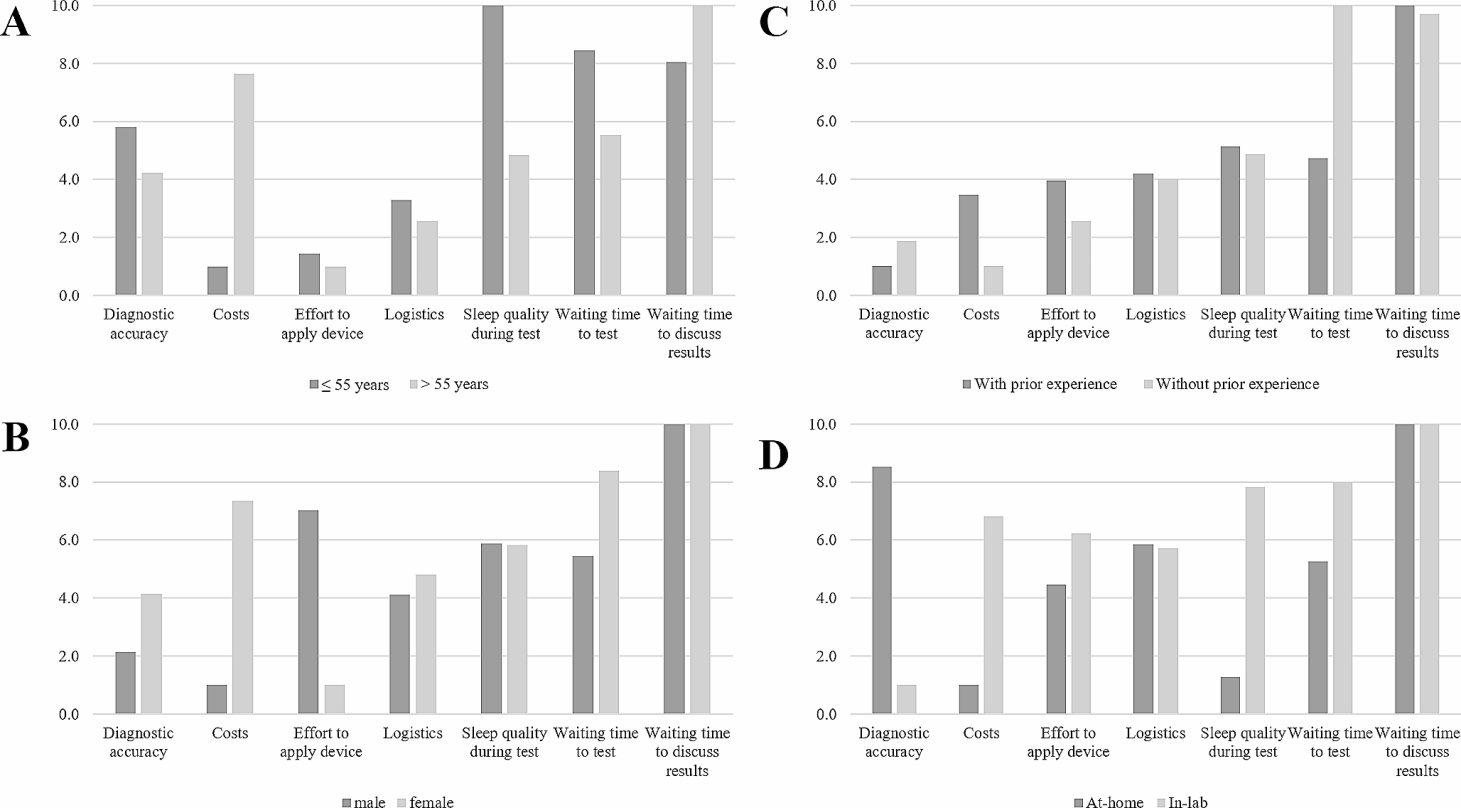
Subgroup analyses of relative attribute importance, re-scaled from 0 (least important) to 10 (most important) and stratified by age (**A**), gender (**B**), prior sleep testing experience (**C**) and preferred location for sleep testing (**D**)

A relatively high Likelihood ratio test statistic provided evidence for heterogeneity in response behavior, which prompted further analyses (Log Likelihood = 182.8 (df = 14), *p* = < 0.001). To better understand preferences across different subgroups, stratification of choice behavior by age, gender, prior experience, and preferred location for sleep testing was performed. For all parameters, significant differences of preferences were found (Fig. 5). Level coefficients for all subgroup analyses are presented in Tables S1 to S4 in the electronic supplement.

Stratification by age showed the largest differences between younger (≤ 55 years old) and older participants (> 55 years old) in the relevance of the attributes *Costs* (0.689 versus 0.209), *Sleep quality* (1.486 versus 0.434), and *Waiting time to test* (1.119 versus 0.677).

Between patients with and without prior experience, the attributes *Waiting time to test* (0.778 versus 2.364) and *Costs* (0.652 versus 0.055) showed the largest differences.

Subgroup analysis by gender (male versus female) revealed relevant differences in the importance of the attributes *Costs* (0.318 versus 1.594), *Effort to apply device* (0.980 versus 0.407), and *Waiting time to test* (0.807 versus 1.924).

Finally, substantial variances were found between participants preferring at-home vs. in-lab for their sleep study, with *Diagnostic accuracy* (1.171 versus 0.711), *Sleep quality* (0.450 versus 1.336), and *Costs* (0.422 versus 1.034), showing the largest differences between these groups.

## Discussion

This study is the first to report preferences for overnight at-home sleep testing in a large sample of patients suffering from sleep disorders. As diagnostic assessments of sleep disorders are increasingly shifting to the patients’ home, the findings of this study can help clinicians and policy makers to optimize patient pathways. As shown in earlier studies, patient perceptions and knowledge of sleep measurements are important drivers of adherence to sleep testing protocols, and can hence impact the effectiveness of medical interventions for sleep disorders [18].

While a broad range of different care models exists, most healthcare systems require a prescription from a sleep physician for coverage of an overnight sleep study. Though HSAT are theoretically limited to diagnosis of sleep-related breathing disorders, they are commonly used as a first-line test to rule out sleep apnea before a more comprehensive sleep assessment is performed. This is not only related to coverage constraints, but increasingly also due to significant limitations of in-laboratory PSG capacity, which can delay access and definitive diagnosis. From that perspective, the DCE designed for this study required patients to make decisions they could encounter in real-life situations.

All attributes presented in this study were statistically significant, except for *Diagnostic accuracy*. This was not expected a priori since this attribute is closely related to receiving a precise diagnosis. As visible from the coefficients of this attribute, the differences in the levels presented were probably not designed large enough to create a stronger response. This is likely because the worst level still had an *acceptable* diagnostic accuracy. This may have participants conclude that a test yielding this accuracy level would be sufficient if used under direction of a physician. This also underlines the relevance of the physicians’ role in the diagnostic assessment of sleep disorders and the need for adequate education of the patient on this important topic. Especially with the emergence of consumer healthcare devices and smartphone applications that claim to assess sleep, the value of a high level of diagnostic accuracy of a sleep test, which is properly validated in clinical trials, should be made clear to the patient. This is not only relevant for the assessment of sleep disorders itself, but furthermore to inform the patient about potential negative long-term consequences on their cardiovascular risk profile. With current scientific advancements that demonstrate the benefits of advanced diagnostic tools with greater prognostic value, the relevance of diagnostic accuracy should not be underestimated.

Among the attributes that reached statistical significance, *Waiting time to discuss results* had the largest coefficients and hence the greatest relative importance for participants. This finding underlines the medical need from the patient perspective and the utility derived from a direct personal consultation without delay. The results presented here are in line with a study conducted in Australia that assessed preferences of OSA-care pathways, in which patients expressed strong preferences for short waiting times [19]. *Sleep quality during test* had the second most influence on the choice decisions and was especially important to patients with a preference for overnight assessment in the sleep laboratory. In light of a recent study by Colleli et al., that reported worse sleep for some patients depending on their individual chronotype, clinicians may evaluate both preferences and chronotype to identify the ideal overnight assessment for each patient to ensure that the needs are properly met [20].

Given the complexity of some measurement devices, it is not surprising that patients prefer support over self-application, though *Effort to apply device* was only of moderate relevance in the choice decisions. Interestingly though, the results were independent of prior experience, a parameter that can influence choice behavior significantly as shown previously on sleep therapeutics [21]. Here again, previous studies have shown that usability of the device plays an important role and influences adherence to prescribed overnight sleep testing [18].

In general, a relatively high heterogeneity was found in response behavior, which led to various subgroup analyses. Previous preference studies in the field of sleep disorders observed comparable differences in choice decisions among patients [22–24]. As increasingly recognized, sleep disorders are rarely homogenous and patients with a common diagnosis such as OSA often have different underlying endo- or phenotypes causing the disease [25, 26]. For this study in particular, participants reported a wide range of symptoms and will have received various diagnoses after the sleep study, which may explain the observed heterogeneity in responses and thus preferences. The variances that were found after stratification for age, gender, prior experience and preferred sleep study location, underline the need for an individual approach to diagnosis of sleep disorders. With the increasing prevalence of sleep disorders, balancing the requirements of individual patients on the one hand, and the provision of care on population level on the other hand, represents a major challenge to sleep physicians, policy makers and payers though, which is gaining in importance with innovative and expensive treatments for sleep disorders being developed and marketed.

Even though preferences in this study were heterogeneous, the attributes *Waiting time to discuss results* and *Waiting time to test* stood out as highly relevant across all subgroups. Given that sleep medical services are a scarce resource in most countries, it underlines the need for a more efficient provision of care and urges institutions in this field to provide the right incentives to ensure attractiveness of sleep medicine for younger physicians [27]. Considering the rising demand for sleep diagnostic testing, this is paramount to ensure sustainable access for patients suffering from sleep disorders to enable timely diagnosis and treatment without delay.

Given the burden of in-laboratory sleep testing, which often requires admission to a hospital, it was not expected that a bit less than half of all participants preferred this type of assessment over an at-home sleep study. Though the average age of patients preferring in-laboratory PSG was significantly higher, stratification of DCE data by age did not reveal any significant differences in the attributes that addressed practical considerations of the sleep study, such as *Effort to apply device*. Stratification by preferred sleep study location though revealed some important differences in choice behavior, with strong preferences for short waiting times among patients that preferred in-laboratory test over home. Patients may have expected that the entire diagnostic process is shorter when admitted to a hospital with daily presence of physicians directly in the sleep laboratory.

Overall, the WTP for out-of-pocket spending for sleep testing was high, which underscores the medical need experienced by patients due to symptoms of sleep disorders. Especially considering the relatively good coverage of sleep medical services in Germany, which usually requires no or rather low co-payments, these findings emphasize the value patients assign to sleep testing and consequently good sleep itself. This is also supported by the correlation that was observed between the number of symptoms and WTP, in which patients with more symptoms, and presumably higher need for treatment, reported higher inclination for out-of-pocket payments for sleep testing.

### Study limitations

Some limitations should be noted for this study, which are largely inherent to the methodology of choice analysis with DCE. First, the attributes of overnight sleep testing that were presented to participants are not exhaustive, and other attributes might will likely also be important for patients. To not overwhelm patients by creating highly complex choice tasks, the list of attributes had to be limited to those seven presented, which is already on the upper end of what is considered appropriate for a DCE. Furthermore, the results of a DCE should be interpreted in the context of the local healthcare system, values as well as cultural background of the participants. By recruiting patients from two sleep centers, based in two states with different sociodemographic backgrounds, the study tried to optimize potential influence from these factors.

Another aspect is that all participants had previous experiences with healthcare services, the majority also with sleep diagnostic procedures. These experiences will have influenced choice behavior and thus the results from the DCE. It was attempted to mitigate this aspect by gathering data on their diagnostic experiences. Due to the length of the questionnaire, and in order not to overwhelm participants, certain information had to be excluded, such as previous or actual waiting times for healthcare services that patients might have experienced. The same holds true for additional medical or demographic information, such as socio-economic status, type of health insurance or education, which may influence decision making. The influence of these factors shall be subject of future studies on this topic.

A further limitation arises from using a stated-preferences over a revealed-preferences approach, which would have been able to evaluate actual choices of patients in need of overnight sleep testing. As patients cannot choose freely in most regulated healthcare systems, due to constraints of for example reimbursement fees, coverage policies and limited availability due to prescription requirements, this approach is commonly not practical in eliciting preferences. Different studies, also in the context of sleep medicine, have shown though, that the stated-preference approach as used in DCE, has a high external validity and can predict choice behavior in real-world situations [11, 12, 28].

## Conclusion

To the authors’ knowledge, this study is the first to report patient preferences for overnight at-home sleep testing, a diagnostic tool increasingly utilized for the assessment of sleep disorders. In this study, patients preferred short waiting times for both, the test itself and the following discussion with the physician, as well as good sleep quality during the measurement. Of lower relevance were the effort to apply the diagnostic device as well as the diagnostic accuracy of the test. This finding underlines the need of patient education by their sleep physician, which need to ensure that appropriate technology with sufficient diagnostic accuracy is selected, which is increasingly important given the emergence of consumer health devices that claim to measure sleep. Overall, substantial heterogeneity in choice behavior was found among study participants, which underlines the non-uniformity of sleep disorders and the importance of personalized care pathways. Health care institutions, payers and providers are urged to investigate and consider preferences when designing sleep medical services, to not only meet the increasing demand for overnight at-home sleep testing, but also ensure patient-centricity in the provision of these services.

## Electronic supplementary material

Below is the link to the electronic supplementary material.

Supplementary Material 1

## Data Availability

The data that support the findings of this study are available from the corresponding author, M.B., upon reasonable request.

## References

[1] Medic G, Wille M, Hemels ME (2017) Short- and long-term health consequences of sleep disruption. Nat Sci Sleep 9:151–161. 10.2147/NSS.S134864 10.2147/NSS.S134864 PMC5449130 28579842

[2] Fietze I et al (2018) Prevalence and association analysis of obstructive sleep apnea with gender and age differences - results of SHIP-Trend. J Sleep Res e12770. 10.1111/jsr.12770 10.1111/jsr.12770 30272383

[3] Aernout E et al (2021) International study of the prevalence and factors associated with insomnia in the general population. Sleep Med 82:186–192. 10.1016/j.sleep.2021.03.028 10.1016/j.sleep.2021.03.028 33957414

[4] Ohayon MM (2011) Epidemiological overview of sleep disorders in the general population. Sleep Med Res 2(1):1–9. 10.17241/smr.2011.2.1.1

[5] Chiao W, Durr ML (2017) Trends in sleep studies performed for medicare beneficiaries. Laryngoscope 127(12):2891–2896. 10.1002/lary.26736 28626986 10.1002/lary.26736

[6] Rosen IM et al Clinical use of a home sleep apnea test: an American Academy of Sleep Medicine position statement. J Clin Sleep Med 13(10):1205–1207. 10.5664/jcsm.6774 10.5664/jcsm.6774 PMC5612637 28942762

[7] Weaver FM et al (2020) Comparing VA and community-based care: trends in sleep studies following the veterans choice act. J Gen Intern Med 35(9):2593–2599. 10.1007/s11606-020-05802-5 10.1007/s11606-020-05802-5 PMC7459009 32242312

[8] Aalaei S et al (2020) Factors affecting patients’ adherence to continuous positive airway pressure therapy for obstructive sleep apnea disorder: a multi-method approach. Iran J Med Sci 45(3):170–178. 10.30476/ijms.2019.45785 10.30476/ijms.2019.45785 PMC7253489 32546883

[9] Samuelson PA (1948) Consumption theory in terms of revealed preference. Economica 15(60):243–253. 10.2307/2549561

[10] Viney R, Lancsar E, Louviere J (2002) Discrete choice experiments to measure consumer preferences for health and healthcare. Expert Rev Pharmacoecon Outcomes Res 2(4):319–326. 10.1586/14737167.2.4.319 10.1586/14737167.2.4.319 19807438

[11] de Bekker-Grob EW et al (2019) Are healthcare choices predictable? The impact of discrete choice experiment designs and models. Value Health J Int Soc Pharmacoeconomics Outcomes Res 22(9):1050–1062. 10.1016/j.jval.2019.04.1924 10.1016/j.jval.2019.04.1924 31511182

[12] Krucien N, Gafni A, Pelletier-Fleury N (2015) Empirical testing of the external validity of a discrete choice experiment to determine preferred treatment option: the case of sleep apnea. Health Econ 24(8):951–965. 10.1002/hec.3076 10.1002/hec.3076 24986760

[13] Bridges JFP et al (2011) Conjoint analysis applications in health—a checklist: a report of the ISPOR good research practices for conjoint analysis task force. Value Health 14(4):403–413. 10.1016/j.jval.2010.11.013 10.1016/j.jval.2010.11.013 21669364

[14] Hauber AB et al (2016) Statistical methods for the analysis of discrete choice experiments: a report of the ISPOR conjoint analysis good research practices task force. Value Health 19(4):300–315. 10.1016/j.jval.2016.04.004 10.1016/j.jval.2016.04.004 27325321

[15] Reed Johnson F et al (2013) Constructing experimental designs for discrete-choice experiments: report of the ISPOR conjoint analysis experimental design good research practices task force. Value Health 16(1):3–13. 10.1016/j.jval.2012.08.2223 10.1016/j.jval.2012.08.2223 23337210

[16] McFadden D (1973) Conditional logit analysis of qualitative choice behavior.

[17] Lancsar E, Fiebig DG, Hole AR (2017) Discrete choice experiments: a guide to model specification. Estimation and Software PharmacoEconomics 35(7):697–716. 10.1007/s40273-017-0506-4 10.1007/s40273-017-0506-4 28374325

[18] Aalaei S et al (2021) Adherence to prescribed overnight sleep study in patients suspected of sleep apnea: problem size and influential factors. Sleep Breath 25(3):1359–1368. 10.1007/s11325-020-02216-9 10.1007/s11325-020-02216-9 33159648

[19] Natsky AN, Vakulin A, Chai-Coetzer CL, McEvoy RD, Adams RJ, Kaambwa B (2022) Preferred attributes of care pathways for obstructive sleep apnoea from the perspective of diagnosed patients and high-risk individuals: a discrete choice experiment. Appl Health Econ Health Policy 20(4):597–607. 10.1007/s40258-022-00716-1 35141851 10.1007/s40258-022-00716-1 PMC9206920

[20] Colelli DR, Dela Cruz GR, Kendzerska T, Murray BJ, Boulos MI (2023) Impact of sleep chronotype on in-laboratory polysomnography parameters. J Sleep Res 32(5):e13922. 10.1111/jsr.13922 37150591 10.1111/jsr.13922

[21] Braun M, Dietz-Terjung S, Taube C, Schoebel C (2022) Treatment preferences and willingness to pay in patients with obstructive sleep apnea: relevance of treatment experience. Somnologie 26(1):1–11. 10.1007/s11818-021-00331-7 34785988 10.1007/s11818-021-00331-7 PMC8579724

[22] Braun M, Dietz-Terjung S, Taube C, Schoebel C (2022) Patient preferences in obstructive sleep apnea—a discrete choice experiment. Sleep Breath. 10.1007/s11325-021-02549-z 10.1007/s11325-021-02549-z 35001351

[23] Krucien N, Gafni A, Fleury B, Pelletier-Fleury N (2013) Patients’ with obstructive sleep apnoea syndrome (OSAS) preferences and demand for treatment: a discrete choice experiment. Thorax 68(5):487–488. 10.1136/thoraxjnl-2012-202240 10.1136/thoraxjnl-2012-202240 PMC3625824 23002172

[24] Krucien N, Le Vaillant M, Pelletier-Fleury N (2015) What are the patients’ preferences for the chronic care model? An application to the obstructive sleep apnoea syndrome. Health Expect Int J Public Particip Health Care Health Policy 18(6):2536–2548. 10.1111/hex.12222 10.1111/hex.12222 PMC5810656 24948135

[25] Bailly S et al (2021) Clusters of sleep apnoea phenotypes: a large pan-European study from the European sleep apnoea database (ESADA). Respirol Carlton Vic 26(4):378–387. 10.1111/resp.13969 10.1111/resp.13969 33140467

[26] Dutta R et al (2021) A novel model to estimate key obstructive sleep apnea endotypes from standard polysomnography and clinical data and their contribution to obstructive sleep apnea severity. Ann Am Thorac Soc 18(4):656–667. 10.1513/AnnalsATS.202001-064OC 10.1513/AnnalsATS.202001-064OC PMC8008997 33064953

[27] Stuck BA, Schöbel C, Spiegelhalder K (2023) Die schlafmedizinische Versorgung in Deutschland. Somnologie 27(1):36–44. 10.1007/s11818-022-00345-9

[28] Quaife M, Terris-Prestholt F, Di Tanna GL, Vickerman P (2018) How well do discrete choice experiments predict health choices? A systematic review and meta-analysis of external validity. Eur J Health Econ 19(8):1053–1066. 10.1007/s10198-018-0954-6 10.1007/s10198-018-0954-6 29380229

