# Supplementary material for: Stated patient preferences for overnight at-home diagnostic assessment of sleep disorders
Source: Sleep Breath. 2024 Jun 15;28(5):1939–49. doi: 10.1007/s11325-024-03080-7 (PMC11449966; doi:10.1007/s11325-024-03080-7)
Supplement: Supplementary file 1 — Supplementary Material 1 [file 11325_2024_3080_MOESM1_ESM.docx]

**Supplement to**

**Preferences for at-home diagnostic assessment among patients with sleep disorders**

| **Attribute / level** | **≤ 55 years old** | | **> 55 years old** | | **Δ Level diff.** |
| --- | --- | --- | --- | --- | --- |
|  | **Coeff.** | **Level diff.** | **Coeff.** | **Level diff.** |  |
| *Diagnostic accuracy*  L1 - Very good  L2 - Good  L3 - Acceptable | 0.269  0.210  -0.479 | 0.689 | -0.019  0.114  -0.095 | 0.209 | 0.480 |
| *Costs*  L1 - 0€  L2 - 100€  L3 - 200€ | 0.161  -0.189  -0.028 | 0.217 | 0.381  0.530  -0.911 | 1.441 | 1.658 |
| *Effort to apply device*  L1 - Self-appliable, 5 min.  L2 - Self-appliable, 15 min.  L3 - With help, 15 min. | -0.044  -0.045  0.089 | 0.134 | -0.623  -0.162  0.785 | 0.947 | 0.812 |
| *Logistics*  L1 - Receive via mail, return via mail  L2 - Receive from clinic, return via mail  L3 - Receive from clinic, return to clinic | -0.370  0.523  -0.153 | 0.217 | -0.028  0.442  -0.414 | 0.386 | 0.603 |
| *Sleep quality during test*  L1 - 9 / 10 report good sleep quality  L2 - 7 / 10 report good sleep quality  L3 - 5 / 10 report good sleep quality | 0.106  0.690  -0.796 | 1.486 | -0.048  0.241  -0.193 | 0.434 | 1.051 |
| *Waiting time to test*  L1 - 1 week  L2 - 2 weeks  L3 - 8 weeks | 0.679  -0.168  -0.511 | 1.119 | 0.565  -0.453  -0.112 | 0.677 | 0.513 |
| *Waiting time to discuss results*  L1 - Discussion with physician next morning  L2 - Discussion with physician within 48 hours  L3 - Discussion with physician within 2 weeks | -0.232  0.675  -0.443 | 1.190 | 0.251  1.016  -1.267 | 2.283 | 1.164 |

**Table S1: Differences of level coefficients between patients ≤ 55 years old and > 55 years old**

| **Attribute / level** | **Male** | | **female** | | **Δ Level diff.** |
| --- | --- | --- | --- | --- | --- |
|  | **Coeff.** | **Level diff.** | **Coeff.** | **Level diff.** |  |
| *Diagnostic accuracy*  L1 - Very good  L2 - Good  L3 - Acceptable | 0.178  0.086  -0.264 | 0.442 | 0.021  0.282  -0.303 | 0.585 | 0.143 |
| *Costs*  L1 - 0€  L2 - 100€  L3 - 200€ | 0.170  -0.021  -0.149 | 0.318 | 0.486  0.554  -1.040 | 1.594 | 1.276 |
| *Effort to apply device*  L1 - Self-appliable, 5 min.  L2 - Self-appliable, 15 min.  L3 - With help, 15 min. | -0.401  -0.177  0.578 | 0.980 | -0.186  -0.034  0.221 | 0.407 | 0.572 |
| *Logistics*  L1 - Receive via mail, return via mail  L2 - Receive from clinic, return via mail  L3 - Receive from clinic, return to clinic | -0.203  0.432  -0.229 | 0.660 | -0.290  0.506  -0.216 | 0.796 | 0.136 |
| *Sleep quality during test*  L1 - 9 / 10 report good sleep quality  L2 - 7 / 10 report good sleep quality  L3 - 5 / 10 report good sleep quality | 0.030  0.411  -0.441 | 0.852 | 0.045  0.534  -0.580 | 1.114 | 0.262 |
| *Waiting time to test*  L1 - Discussion with physician next morning  L2 - Discussion with physician within 48 hours  L3 - Discussion with physician within 2 weeks | 0.370  -0.437  0.067 | 0.807 | 1.053  -0.182  -0.871 | 1.924 | 1.117 |
| *Waiting time to discuss results*  L1 - 1 week  L2 - 2 weeks  L3 - 8 weeks | 0.024  0.640  -0.664 | 1.304 | 0.056  1.188  -1.244 | 2.432 | 1.128 |

**Table S2: Differences of level coefficients between male and female patients**

| **Attribute / level** | **With prior experience** | | **Without prior experience** | | **Δ Level diff.** |
| --- | --- | --- | --- | --- | --- |
|  | **Coeff.** | **Level diff.** | **Coeff.** | **Level diff** |  |
| *Diagnostic accuracy*  L1 - Very good  L2 - Good  L3 - Acceptable | 0.126  0.141  -0.267 | 0.408 | -0.051  0.163  -0.112 | 0.275 | 0.133 |
| *Costs*  L1 - 0€  L2 - 100€  L3 - 200€ | 0.252  0.148  -0.400 | 0.652 | -0.016  0.035  -0.020 | 0.055 | 0.597 |
| *Effort to apply device*  L1 - Self-appliable, 5 min.  L2 - Self-appliable, 15 min.  L3 - With help, 15 min. | -0.289  -0.122  0.412 | 0.701 | -0.059  0.257  -0.198 | 0.455 | 0.246 |
| *Logistics*  L1 - Receive via mail, return via mail  L2 - Receive from clinic, return via mail  L3 - Receive from clinic, return to clinic | -0.164  0.444  -0.279 | 0.723 | -0.464  0.104  0.360 | 0.824 | 0.101 |
| *Sleep quality during test*  L1 - 9 / 10 report good sleep quality  L2 - 7 / 10 report good sleep quality  L3 - 5 / 10 report good sleep quality | 0.048  0.385  -0.433 | 0.818 | -0.178  0.614  -0.436 | 1.050 | 0.232 |
| *Waiting time to test*  L1 - Discussion with physician next morning  L2 - Discussion with physician within 48 hours  L3 - Discussion with physician within 2 weeks | 0.464  -0.314  -0.150 | 0.778 | 1.116  0.132  -1.248 | 2.364 | 1.586 |
| *Waiting time to discuss results*  L1 - 1 week  L2 - 2 weeks  L3 - 8 weeks | 0.021  0.638  -0.660 | 1.298 | -0.227  1.258  -1.032 | 2.290 | 0.992 |

**Table S3: Differences of level coefficients between patients with and without prior sleep testing experience**

| **Attribute / level** | **Preferred test location at-home** | | **Preferred test location in-laboratory** | | **Δ Level diff.** |
| --- | --- | --- | --- | --- | --- |
|  | **Coeff.** | **Level diff.** | **Coeff.** | **Level diff** |  |
| *Diagnostic accuracy*  L1 - Very good  L2 - Good  L3 - Acceptable | 0.325  0.522  -0.847 | 1.171 | -0.112  -0.299  0.412 | 0.711 | 1.882 |
| *Costs*  L1 - 0€  L2 - 100€  L3 - 200€ | 0.248  -0.073  -0.175 | 0.422 | 0.289  0.456  -0.745 | 1.034 | 0.612 |
| *Effort to apply device*  L1 - Self-appliable, 5 min.  L2 - Self-appliable, 15 min.  L3 - With help, 15 min. | -0.303  -0.161  0.465 | 0.768 | -0.397  -0.061  0.458 | 0.855 | 0.087 |
| *Logistics*  L1 - Receive via mail, return via mail  L2 - Receive from clinic, return via mail  L3 - Receive from clinic, return to clinic | -0.143  0.524  -0.381 | 0.905 | -0.313  0.386  -0.072 | 0.699 | 0.205 |
| *Sleep quality during test*  L1 - 9 / 10 report good sleep quality  L2 - 7 / 10 report good sleep quality  L3 - 5 / 10 report good sleep quality | -0.185  0.265  -0.080 | 0.450 | 0.321  0.694  -1.015 | 1.336 | 0.885 |
| *Waiting time to test*  L1 - Discussion with physician next morning  L2 - Discussion with physician within 48 hours  L3 - Discussion with physician within 2 weeks | 0.460  -0.387  -0.073 | 0.847 | 0.822  -0.264  -0.558 | 1.381 | 0.534 |
| *Waiting time to discuss results*  L1 - 1 week  L2 - 2 weeks  L3 - 8 weeks | -0.117  0.718  -0.601 | 1.319 | 0.147  0.919  -1.067 | 1.986 | 0.667 |

**Table S4: Differences of level coefficients between patients preferring at-home sleep testing versus preferring in-laboratory sleep testing**
